# Can education change the world? Education amplifies differences in liberalization values and innovation between developed and developing countries

**DOI:** 10.1371/journal.pone.0199560

**Published:** 2018-06-21

**Authors:** Alain Van Hiel, Jasper Van Assche, David De Cremer, Emma Onraet, Dries Bostyn, Tessa Haesevoets, Arne Roets

**Affiliations:** 1 Ghent University, Department of Developmental, Personality and Social Psychology, Henri Dunantlaan 2, Gent, Belgium; 2 University of Cambridge, Judge Business School, Trumpington Street, Cambridge, United Kingdom; Universidad Veracruzana, MEXICO

## Abstract

The present study investigated the relationship between level of education and liberalization values in large, representative samples administered in 96 countries around the world (total *N* = 139,991). These countries show meaningful variation in terms of the Human Development Index (HDI), ranging from very poor, developing countries to prosperous, developed countries. We found evidence of cross-level interactions, consistently showing that individuals’ level of education was associated with an increase in their liberalization values in higher HDI societies, whereas this relationship was curbed in lower HDI countries. This enhanced liberalization mindset of individuals in high HDI countries, in turn, was related to better scores on national indices of innovation. We conclude that this ‘education amplification effect’ widens the gap between lower and higher HDI countries in terms of liberalized mentality and economic growth potential. Policy implications for how low HDI countries can counter this gap are discussed.

## Introduction

Education increases human capital and, in turn, innovation and economic growth [1, 2]. However, because educational systems are more successful in creating human capital in developed countries than in less prosperous countries [3, 4], participation in education contributes to the economic differences between these countries. Education not only fosters the acquisition of skills and knowledge, but also sustains liberalization values in the form of autonomy and personal freedom [5, 6]. The goal of the present study is to uncover the role of this ‘liberalizing’ effect of education in the impact of education on economic differences among countries. Specifically, we aim to show that as individuals participate more in education, they develop a more liberalized mindset, which in turn increases a country’s innovation potential. However, we hypothesize that also in this respect the benefits are greater in developed countries than in developing countries.

A classic study on the liberalizing effect of education was conducted in the 1930s by the famous social psychologist Theodor Newcomb [7, 8]. At Bennington College in Vermont, he measured students’ attitudes each year until graduation. Most students attending this college came from conservative families. However, the school cultivated a liberal atmosphere, in the spirit of President F. D. Roosevelt’s New Deal. The study showed that the students’ attitudes gradually shifted toward more openness and a liberalized mindset, a shift that even persisted until in their sixties and seventies [9]. Other studies reported similar relationships between educational level and liberalization values in North America and Western Europe [10]. Education thus not only transmits skills and knowledge, but it also represents a vehicle for the dissemination and transmission of particular worldviews. A yet unanswered question, however, is whether the liberalizing effect of education also emerges (as strongly) in other parts of the world, like in less developed countries.

Countries show very different levels of development. These differences do not only pertain to economic development, but also to the strength of public services. Indicators of income, health and education constitute the so-called Human Development Index (HDI), a measure of development that combines economic prosperity and well-being [11]. The highest-ranking countries in terms of HDI include countries like Norway, Canada and Japan, whereas the lowest-ranking countries include African countries like Niger and Sierra Leone. The online appendix (S1 File) contains the HDI scores of the countries included in the present study. These prominent differences among countries in HDI levels, in turn, may have important consequences for how a country’s citizens perceive the social world.

Inhabitants of poorly developed countries (countries with a low HDI) generally tend to endorse a more closed, conservative worldview with an emphasis on traditional values and economic and physical security, whereas citizens of higher HDI societies typically endorse a more open-minded, liberal worldview, emphasizing freedom, individualization and self-expression [12, 13, 14]. We refer to these values in terms of liberalization values. Individuals are heavily influenced by the values of others around them [15] and as a result, the presence of people endorsing particular values in terms of less or more liberalization constitutes a value climate which serves as a guiding norm. Sociologists have coined these climates as ‘cultural beliefs’ and have constructed a ‘cultural map’ of the world which positions countries in terms of their value climate [12, 13, 14]. Because a liberalized climate is a context par excellence for education to increase such values among its students [7, 8, 9, 10], we hypothesize that participation in education has a stronger liberalizing effect on citizens of higher HDI countries compared to citizens of lower HDI countries (Hypothesis 1).

Sociological studies already have shown that the development of a liberalized climate is a basis for the transition from industrialized to post-industrialized, innovative and knowledge-based societies [12, 13, 14]. However, in the present study, we are particularly interested in how participation in education changes the mind of the individual. In psychological literature, it is an established finding that conservative, closed-minded people are oriented toward the preservation of the status quo and that they often tend to oppose change, whereas liberal people often judge change to be necessary [16]. There is also ample evidence that conservative individuals tend to be more closed-minded, prefer clear structures, and lack openness [17, 18]. Such mindsets clearly do not constitute a sound psychological basis for innovation to occur [19, 20]. The hypothesized changes in terms of liberalized mindset of individuals may add up and eventually increase the innovation potential of their country. The liberalizing effect of education, which we expect to be particularly present in higher HDI countries, should thus enlarge a population’s pool of individuals who are able and equipped to promote innovation. For these reasons, Hypothesis 2 states a mediated moderation effect in which the interaction effect between education and HDI on innovation works through increased liberalization.

## Method

The present study included data from representative samples administered in Wave 5 (2005–2009) and 6 (2010–2014) of the World Values Survey (WVS), and the 2008 Wave of the European Values Study (EVS), which use almost identical questionnaires and methodologies [21, 22]. Our final data included 139,991 participants from 96 samples from different countries across six continents. We included educational level and liberalization values as individual-level measures. We used two indicators of education level (for details, see S1 File). To measure liberalization values, we computed an aggregated scale based on the items that have been used in multiple WVS rounds to construct the Inglehart-Welzel cultural map of the world [12, 13, 14] (for details, see S1 File). We also distinguished between two bipolar components that constitute liberalized mentality. Secular-rational values give high priority to freedom as opposed to traditional values that emphasize authority, religion, and family values. Self-expression or emancipative values give high priority to individualization under the form of self-expression, subjective well-being and quality of life as opposed to survival values that place emphasis on economic and physical security. The analyses of these two distinct components of liberalization yielded results similar to the aggregated scale and can be found in S1 File.

Country-level measures were Human Development Index [11] (for details, see S1 File) and Innovation. In order to operationalize a country’s innovation level, the present study used four well-accepted indicators of countries’ capacity for and success in innovation that are considered important benchmarks for policymakers and business leaders: The Global Innovation Index [23], the Global Competitiveness Index [24], the Innovation Capacity Index [25], and the International Innovation Index [26]. A description of the methods can be found in the online appendix (S1 File).

## Results

### Preliminary analyses

Because participants (individual level) were nested within countries (contextual level), we first investigated an empty (intercept-only) model. The intraclass coefficients (ICC) was large (.46), indicating that there is substantial between-level variance in the dependent measure, warranting the use of a multi-level analyses. All independent variables were grand-mean centered [27], and we used full-information maximum-likelihood estimates with robust standard errors. For none of our variables was the proportion of missing values higher than 0.4%. The correlation between education and liberalization values was significant, *r* = .28, P< .001, as well as the correlation between liberalization values and innovation, *r* = .81, P< .001.

### Main analysis

**Fig 1:**
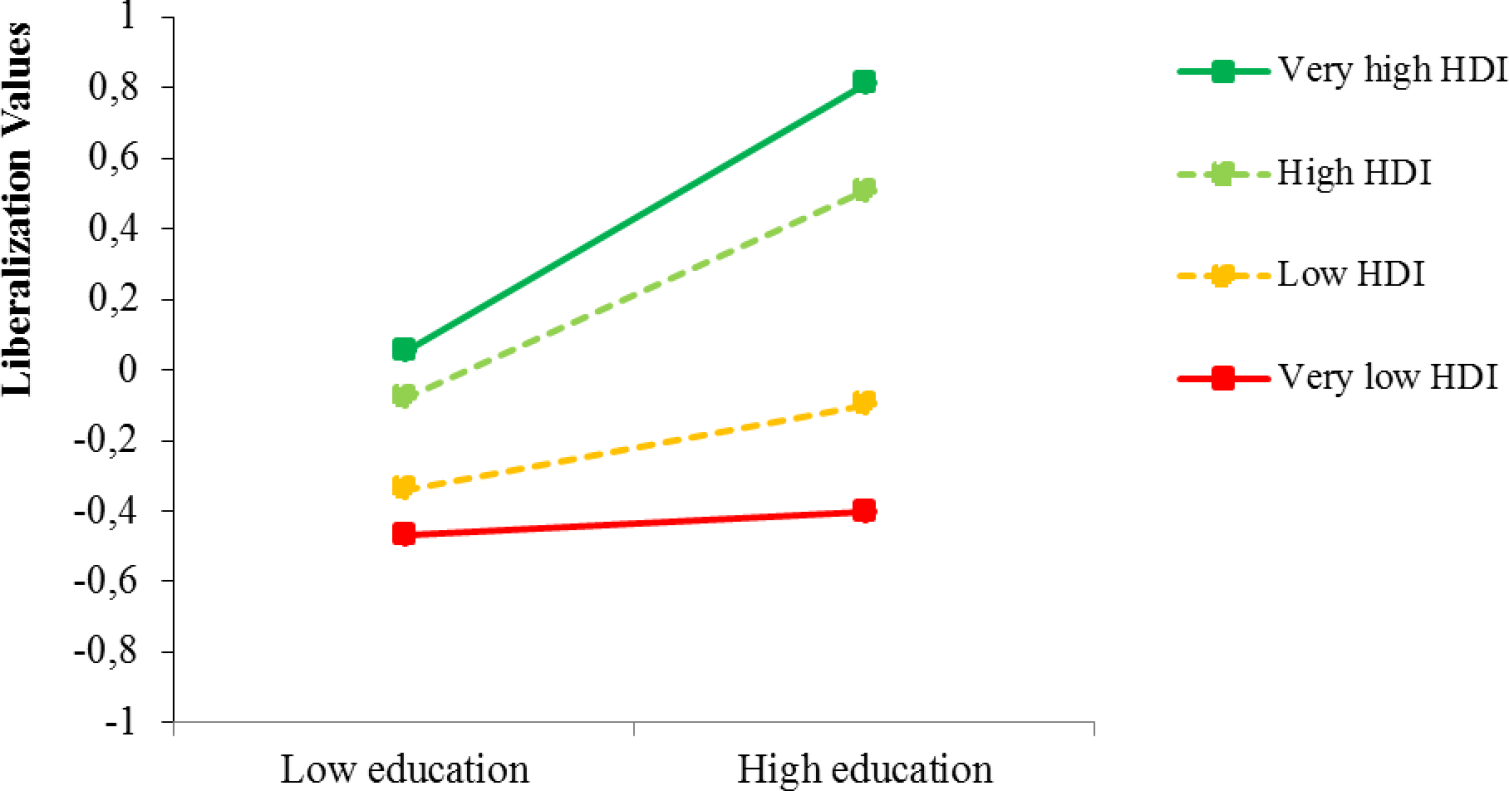
Cross-level interactions between education and the human development index on liberalization values.

A multilevel model (MLM) based on a *random coefficient model* [27] was conducted. We explored the variance in the slopes, testing differences in the relation between education and liberalized mindset across countries. Inclusion of gender, age, and religiosity in the first step of the multilevel regression did not change the pattern of results. For the sake of parsimony, we only report the results without background variables. As shown in Table 1 and in line with previous studies, education is positively related to liberalization values and individuals in higher HDI countries show higher overall levels of liberalization values than individuals in lower HDI countries. Moreover, consistent with Hypothesis 1, stronger relationships between education and liberalization values emerged in higher HDI countries compared to lower HDI countries. Multilevel simple slope analyses (Fig 1) revealed that in countries with a very low HDI (2 SD below the mean), education was not significantly related to liberalized mindset [*b* = 0.02 (SE = 0.01), *β* = 0.03, P = .20]. The association was somewhat stronger in countries with HDI levels 1 SD below the mean [*b* = 0.07 (SE = 0.01), *β* = 0.12, P< .001]. In countries with a high (1 SD above the mean) and an extremely high (2 SD above the mean) HDI, the magnitude of the relationship between education level and liberalization values further increased [*b* = 0.16 (SE = 0.01), *β* = 0.30, P< .001, and *b* = 0.21 (SE = 0.01), *β* = 0.39, P< .001, respectively]. Detailed analyses also revealed a significant interaction effect between country level HDI and individual-level education on country-level innovation (*b* = 4.11 (SE = 0.57), *β* = 0.60, P< .001). Again, the association between education and innovation was stronger in high HDI countries.

**Table 1:** Unstandardized (standard errors in brackets) and standardized estimates of multilevel regression analyses, examining the moderating role of the human development Index (HDI) in the associations of education with liberalization values (based on *N* = 139,991) and with innovation (based on *N* = 96).

	Liberalization values		Innovation	
	*b* (SE)	*β*	*b* (SE)	*β*
Education	0.11 (0.01)	0.20***	0.19 (0.14)	0.18
HDI	1.54 (0.21)	0.44***	6.25 (0.55)	0.89***
Education X HDI	0.31 (0.05)	0.09***	4.11 (0.57)	0.60***

***: P< .001

**Fig 2:**
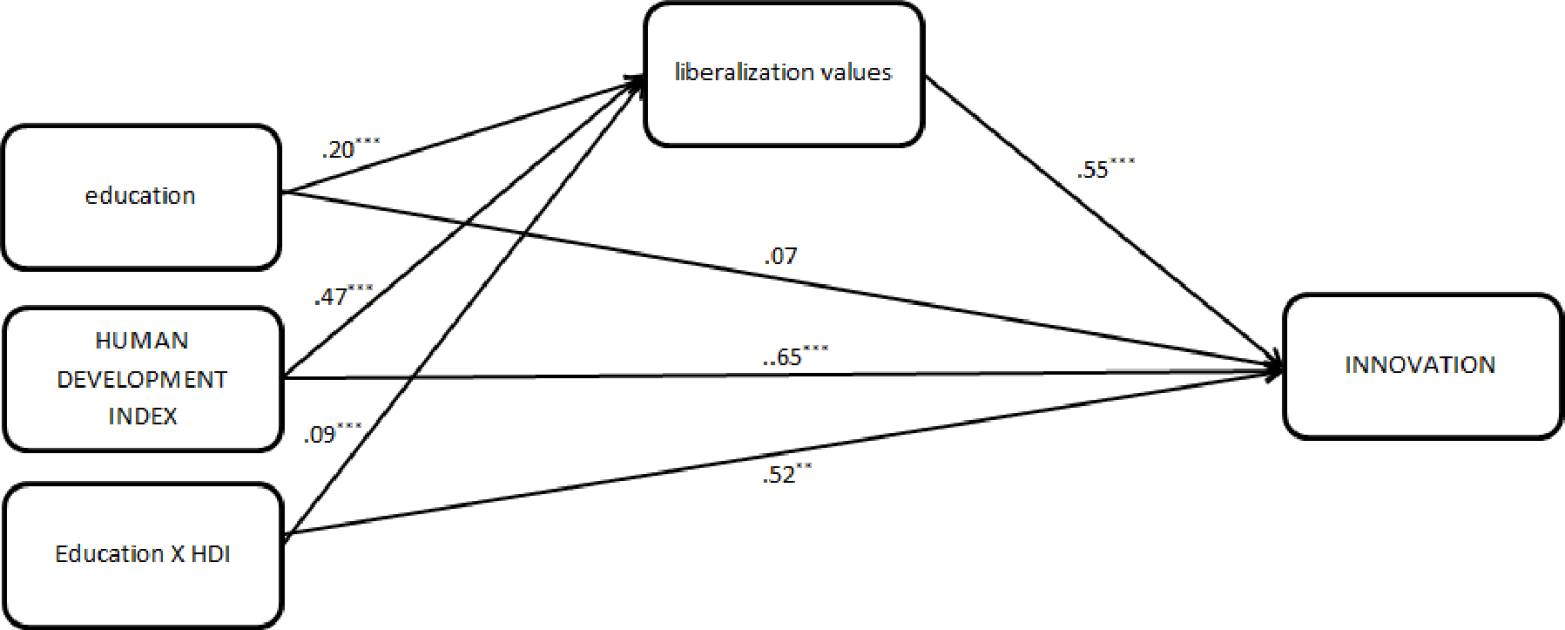
Standardized estimates of multilevel path model regressing country-level innovation on individual-level education, country-level HDI, their cross-level interaction, and individual-level liberalization values. Note: Variables in capital letters represent country-level variables. ***: P< .001,** P< .01.

Next, a mediational multilevel path model was examined to test whether individual-level education and country-level HDI relate to country-level innovation via individual-level liberalization values (Fig 2) (for the code in MPlus, see S1 File). Consistent with Hypothesis 2, the cross-level interaction between education and HDI on innovation via liberalization values was significant [*b* = 0.35 (SE = 0.08), *β* = 0.05, *P* < .001]. In other words, the liberalizing effect of education (which is particularly strong in higher HDI countries) further relates to higher country-level innovation.

### Subsidiary analysis

Although these analyses confirm our Hypotheses, it should be acknowledged that previous studies have revealed that education increases human capital which leads to innovation [1, 2], especially in developed countries [3, 4]. We thus further tested the mediational multilevel model controlling for levels of human capital. Unfortunately, EVS and WVS do not contain measures of human capital, and this variable could therefore not be included at the individual level. Instead, a national indicator of human capital was included as a control variable. This indicator consisted of the country-level test scores on the standardized PISA test of mathematics, reading abilities and scientific knowledge, administered among 15-year olds. We obtained scores from 62 countries (total number of participants = 672,028, see S1 File) which participated in the 2009 PISA wave [28]. The analyses controlling for country-level human capital revealed that indirect effect of the cross-level interaction between education and HDI on innovation via liberalization values remained significant [*b* = 0.24 (SE = 0.12), *β* = 0.04, P = .04].

## Discussion

The results of this study show that an individual’s level of education is more strongly related to having a liberalized mindset in higher HDI compared to lower HDI countries. Moreover, through this increased liberalization, education resulted in higher innovation ratings in higher HDI countries. We draw two conclusions. First, education amplifies the differences between the mindset of citizens in highly developed countries who increasingly liberalize, and the mindset of citizens in poorly developed countries who remain as closed-minded and conservative as before, or only show a small increase in liberalization values. This trend is likely to become even more pronounced in the future. Indeed, because education is widely available in Western countries, it will further lead to a shift in the mentality of a large part of the population in the direction of liberalization values. In less developed countries, on the contrary, the small group of highly educated people who only shift a small degree in the liberalized direction (or, fail to show such a shift in the lowest HDI countries) will have too little influence to shift a given country’s mentality.

A second conclusion pertains to the effects of education on innovation, and as a result, on the potential for economic prosperity in the long run. The widening gap among countries in terms of mentality has important indirect effects on prosperity as well. Whereas a liberalized mindset has been typically studied in sociology, political psychology and political science, it has not yet been fully considered in previous studies that investigated the relationship between education and innovation and, therefore, economic growth. This is a notable omission. As already shown in literature, educational systems are more successful in creating human capital in developed countries compared to developing countries [3, 4]. As such, they may differentially contribute to innovation and thus widen the economic gap between these countries. The present study shows that education significantly contributes to this widening gap in yet another way. Specifically, the potential of education to increase liberalization in developed countries creates a sound psychological basis for innovation to occur [19, 20].

A limitation of the present study is the correlational design that does not allow for a firm conclusion about the direction of the effects. The availability of more waves of worldwide surveys and tests in the future will undoubtedly create opportunities for scholars to study the present relationships in a longitudinal design. Such a study would allow investigating the presence of reversed paths. It is indeed plausible that a high level of innovation in a given country spurs the development of liberalization values, and that a high level of liberalization values prompts governments to increase the supply of education. Along similar lines, innovative activity brings prosperity that enables governments to invest additional resources in educational systems. Hence, a model with recursive paths in which education leads to liberalization values and innovation, and liberalization values and innovation in turn promote education, is possible. These recursive paths may strengthen each other and constitute an upward spiral, which sustains the development of low HDI countries.

The present study has policy implications especially for low HDI countries. Such countries would undoubtedly benefit of educational reforms that support ‘human capital’ in the form of the acquisition of skills and knowledge [3, 4]. However, at the same time, such reforms should also target the development of liberalization values, which can materialize by providing students with an autonomy-sustaining class climate [5]. Such reforms–which do not pose any financial burden—promote personal freedom and autonomy [6], or put otherwise, liberalization. Conversely, when classroom conditions stress control, demands, and performance-contingent rewards, both the quality and reach of education is impoverished, and the liberalization process is burdened. As we argued in the preceding paragraph, policy makers could also try to bring in to motion the education-liberalization circle by investments in innovative activity, which in turn may free up financial means to invest in education. However, there are huge differences among countries in the efficiency of investments in innovation, which are not well understood yet and call for further investigation [29]. On basis of the available evidence it can therefore be concluded that, especially in low HDI countries, policies that promote liberalization in educational settings is a cost efficient (or even costless) tool by which inhabitants of a country develop the ‘suitable mindset’ for innovation to occur, and that expenditures in innovative activities are best accompanied by such liberalization sustaining policies. This could allow low HDI countries to break the vicious circle of underdevelopment.

In conclusion, our findings indicate that education has quite different effects on the pace of liberalization, which in turn amplifies the cultural and economic gap between developed and developing countries. So, if “education can change the world”, then this seems to be particularly true for developed countries as opposed to developing countries, as long as the educational system in the latter countries does not foster liberalization values.

## Supporting information

S1 File Online appendix. (DOCX)
