# Supplementary material for: Can education change the world? Education amplifies differences in liberalization values and innovation between developed and developing countries
Source: PLoS One. 2018 Jun 21;13(6):e0199560. doi: 10.1371/journal.pone.0199560 (PMC6013109; doi:10.1371/journal.pone.0199560)
Supplement: S1 File — (DOCX) [file pone.0199560.s001.docx]

**Online Appendix**

**Description of measures**

**Individual-level measures**

**Education**

We used two indicators of education level. The WVS [1] assessed an individual’s highest level of education with a 9-point Likert scale from 1 (“no formal education”) to 9 (“university-level education, with degree”; *M*= 5.56, *SD*= 2.45). The EVS [2] used the International Standard Classification of Education (ISCED), ranging from 0 (“pre-primary education or no education”) to 6 (“second stage of tertiary education”; *M*= 3.07, *SD*= 1.32). Both databases also asked participants at what age they completed their full-time education, either at school or at an institution of higher education (excluding apprenticeships; *M*= 19.56, *SD*= 6.96). All indicators were *z*-standardized and averaged before composing a scale. The correlation between the two indicators was strong and positive in both the WVS and the EVS (*r* = 0.45 and 0.70, P*’*s< .001, respectively).

***Liberalization values***

To measure the values dimensions, we selected the items that have been used in multiple WVS rounds to construct the Inglehart-Welzel cultural map of the world [3-5]. Because we were interested in the potential of education to increase liberalization values, we selected the five items that tap into the preference for secular-rational, as opposed to traditional, values, as well as the five items that probe into self-expression (vs. survival) values^5^. The first item, “God is very important in my life,” was rated on a 10-point Likert scale ranging from 1 (“not at all important”) to 10 (“very important). The second indicator was computed via the 4-item Autonomy Index, where lower scores (-2) indicate that a person believes that it is more important for a child to learn obedience and religious faith than to learn independence and determination (+2). For the third item, participants had to specify if “abortion is never justifiable” on a 10-point Likert scale ranging from 1 (“never justifiable”) to 10 (“always justifiable”). The fourth item, “I have a strong sense of national pride,” was rated on a 4-point Likert scale ranging from 1 (“very proud”) to 4 (“not at all proud”), and the fifth item asked if “respect for authority would be a…” (1 = “good thing”; 2 = “don’t mind”; 3 = “bad thing”). The sixth item was computed based on the 4-item Materialist/Postmaterialist Values Index where a score of 1 represents a priority on economic and physical security, and a score of 3 represents a priority of self-expression and quality of life. The seventh item reads “Taking all things together, would you say you are…” (1 = “very happy”; 2 = “rather happy”; 3 = “not very happy”; 4 = “not at all happy”). For the eighth item, participants had to specify if “homosexuality is never justifiable” on a 10-point Likert scale ranging from 1 (“never justifiable”) to 10 (“always justifiable”). The ninth item, “signing a petition,” was scored on a 3-point scale (1 = “have done”; 2 = “might do”; 3 = “would never do”), and for the last item, participants had to select either 1 (“Most people can be trusted”) or 2 (“You have to be very careful about trusting people”).

Item 1, 7, 9 and 10 were reverse coded, and we *z*-standardized all items before computing a scale. The aggregated liberal values scale showed an acceptable internal consistency, Cronbach’s α = .65. The scale’s internal consistency is below the recommended threshold of .70 [6], which indicates that the scale is rather polymorphous. This relatively low level of internal consistency may have curbed the relationships of liberalization values with the other study’s variables.”

**Country-level measures**

**Human Development Index**

The Human Development Index (HDI) is a composite statistic of life expectancy, education, and income indices used to rank countries into four tiers of human development [7]. We used statistics from the year of data collection in the respective EVS/WVS wave (2005, 2008, or 2010; *M* = 0.75, *SD* = 0.14).

**Innovation**

Four well-accepted indicators of innovation were used. These indicators have become important benchmarks for policymakers and business leaders: The Global Innovation Index (*M*= 3.44, *SD*= 0.72, *N*= 84) [8], the Global Competitiveness Index (*M*= 4.39, *SD*= 0.61, *N*= 88) [9], the Innovation Capacity Index (*M*=55.89, *SD*= 11.76, *N* = 81) [10] and the International Innovation Index (*M*= .25, *SD*= 0.98, *N*= 78) [11]. These indicators were used to analyze countries’ capacity for and success in innovation. We *z*-standardized the four indices, which we found to be strongly interrelated (.90 < *r*’s < .96). The scale was calculated by taking the mean of the available indices and showed very high internal consistency, Cronbach’s α = .98.

**Human capital**

The measure of human capital consisted of the country-level test scores on the standardized PISA test on mathematics, reading abilities and scientific knowledge, which has been administered among 15-year olds [12]. This measure was used as a control variable. We selected PISA2009 and PISA2009 Plus, which is the wave has been administered in the largest number of countries. The PISA2009 Plus wave (including data of Georgia, Malaysia, Malta, Moldova, and India) was administered in 2010, using the same tests as the PISA2009 wave. As the human capital indicator of the Indian sample, we used the mean score of the Himachal Pradesh and Tamil Nadu regions. The relationships among the test scores of mathematics (*M*= 460, *SD*= 60), reading abilities (*M*= 457, *SD*= 55) and scientific knowledge (*M*= 464, *SD*= 59) were strong and positive (.95 < *r*’s < .97, *N* = 62). The scale showed very high internal consistency, Cronbach’s α = .99.

**Overview of included countries and scores on country-level HDI, Innovation and human capital**

|  | Wave | HDI | GII | GCI | III | ICI | PISA |
| --- | --- | --- | --- | --- | --- | --- | --- |
| Albania | EVS | 0.703 | 2.86 | 3.94 | -0.49 |  | 384 |
| Algeria | WVS 6 | 0.709 | 2.50 | 3.96 | -0.83 | 42.5 |  |
| Andorra | WVS 5 | 0.932 |  |  |  |  |  |
| Argentina | WVS 5 | 0.758 | 2.91 | 3.95 | -0.97 | 49.3 | 396 |
| Armenia | WVS 6 | 0.720 | 2.84 | 3.76 | -0.66 |  |  |
| Australia | WVS 6 | 0.926 | 4.28 | 5.11 | 1.02 | 69.4 | 519 |
| Austria | EVS | 0.868 | 4.21 | 5.09 | 1.15 | 66.7 | 487 |
| Azerbaijan | WVS 6 | 0.743 | 3.09 | 4.29 | -0.54 | 53.8 | 389 |
| Belarus | WVS 6 | 0.779 |  |  |  |  |  |
| Belgium | EVS | 0.873 | 4.31 | 5.07 | 0.86 | 66.1 | 509 |
| Bosnia | EVS | 0.727 | 2.58 | 3.70 |  | 47.5 |  |
| Brazil | WVS 5 | 0.705 | 2.97 | 4.28 | -0.59 | 45.3 | 390 |
| Bulgaria | EVS | 0.766 | 3.26 | 4.16 | -0.13 | 57.4 | 432 |
| Burkina Faso | WVS 5 | 0.321 | 2.48 | 3.20 | -0.97 |  |  |
| Canada | WVS 5 | 0.892 | 4.55 | 5.30 | 1.42 | 73.6 | 530 |
| Chile | WVS 6 | 0.808 | 3.35 | 4.69 | 0.21 | 58.3 | 439 |
| China | WVS 6 | 0.701 | 3.32 | 4.84 | 0.73 | 49.9 |  |
| Colombia | WVS 6 | 0.706 | 2.76 | 4.14 | -0.66 | 49.4 | 399 |
| Cyprus | WVS 6 | 0.848 | 3.61 | 4.50 | 0.63 | 55.2 |  |
| Czech Republic | EVS | 0.856 | 3.77 | 4.57 | 0.41 | 57.8 | 490 |
| Denmark | EVS | 0.896 | 4.72 | 5.32 | 1.60 | 74.3 | 499 |
| Ecuador | WVS 6 | 0.701 | 2.43 | 3.65 | -1.11 | 44.6 |  |
| Egypt | WVS 6 | 0.678 | 2.91 | 4.00 | -0.47 | 46.6 |  |
| Estonia | WVS 6 | 0.830 | 3.76 | 4.61 | 0.94 | 60.5 | 514 |
| Ethiopia | WVS 5 | 0.339 | 2.46 | 3.51 | -0.72 | 39.2 |  |
| Finland | EVS | 0.878 | 4.66 | 5.37 | 1.87 | 76.1 | 544 |
| France | EVS | 0.875 | 4.20 | 5.13 | 1.12 | 65.3 | 497 |
| Georgia | EVS | 0.730 | 2.83 | 3.86 | -0.75 | 55 | 375 |
| Germany | WVS 6 | 0.904 | 4.32 | 5.39 | 1.12 | 68.9 | 510 |
| Ghana | WVS 6 | 0.556 | 2.66 | 3.56 |  | 46.4 |  |
| Great Britain | WVS 5 | 0.888 | 4.42 | 5.25 | 1.42 | 71.3 | 500 |
| Greece | EVS | 0.858 | 3.28 | 3.99 | 0.12 | 49.9 | 473 |
| Guatemala | WVS 5 | 0.576 | 2.72 | 4.04 | -0.99 | 44.7 |  |
| Hong Kong | WVS 5 | 0.839 | 4.83 | 5.3 | 1.88 | 71.4 | 546 |
| Hungary | EVS | 0.814 | 3.54 | 4.33 | 0.51 | 56.8 | 496 |
| India | WVS 5 | 0.527 | 3.10 | 4.33 | 0.06 | 44.2 | 336 |
| Indonesia | WVS 5 | 0.640 | 2.95 | 4.43 | -0.57 | 46.0 | 385 |
| Iran | WVS 5 | 0.681 |  | 4.14 |  | 44.5 |  |
| Iraq | WVS 6 | 0.638 |  |  |  | 32.6 |  |
| Ireland | EVS | 0.902 | 4.27 | 4.74 | 1.88 | 69.1 | 497 |
| Italy | WVS 5 | 0.858 | 3.47 | 4.37 | 0.21 | 56.7 | 486 |
| Japan | WVS 6 | 0.884 | 4.50 | 5.37 | 1.79 | 70.2 | 529 |
| Jordan | WVS 6 | 0.744 | 3.08 | 4.21 | -0.15 | 53.7 | 402 |
| Kazakhstan | WVS 6 | 0.747 | 3.05 | 4.12 | -0.23 | 53.1 | 398 |
| Kosovo | EVS | 0.822 |  |  |  |  |  |
| Kuwait | WVS 6 | 0.807 | 3.56 | 4.59 | 0.06 | 51.3 |  |
| Kyrgyzstan | WVS 6 | 0.614 | 2.67 | 3.49 | -0.77 |  | 325 |
| Latvia | EVS | 0.813 | 3.29 | 4.14 | 0.12 | 58.7 | 487 |
| Lebanon | WVS 6 | 0.759 |  | 3.89 |  | 44.3 |  |
| Libya | WVS 6 | 0.799 | 2.65 | 3.74 |  |  |  |
| Lithuania | EVS | 0.827 | 3.44 | 4.38 | 0.16 | 59.6 | 479 |
| Luxemburg | EVS | 0.882 | 4.38 | 5.05 | 1.54 | 72.2 | 482 |
| Malaysia | WVS 6 | 0.766 | 3.77 | 4.88 | 1.12 | 56.4 | 413 |
| Mali | WVS 5 | 0.359 | 2.66 | 3.28 |  | 35.0 |  |
| Malta | EVS | 0.809 | 3.74 | 4.34 | 0.20 | 54.6 | 455 |
| Mexico | WVS 6 | 0.748 | 2.96 | 4.19 | -0.16 | 50.2 | 420 |
| Moldova | EVS | 0.652 |  | 3.86 | -0.8 |  | 399 |
| Montenegro | EVS | 0.780 | 3.08 | 4.36 |  |  | 404 |
| Morocco | WVS 6 | 0.603 | 2.74 | 4.08 | -0.57 | 44.2 |  |
| Netherlands | WVS 6 | 0.904 | 4.62 | 5.33 | 1.55 | 72.8 | 519 |
| New Zealand | WVS 6 | 0.903 | 4.60 | 4.92 | 0.77 | 71.3 | 524 |
| Nigeria | WVS 6 | 0.492 | 2.69 | 3.38 | -0.95 | 36.8 |  |
| Northern Ireland | EVS | 0.714 |  |  |  |  |  |
| Norway | WVS 5 | 0.935 | 4.59 | 5.14 | 1.14 | 72.0 | 500 |
| Pakistan | WVS 6 | 0.526 | 2.67 | 3.48 | -0.82 | 40.8 |  |
| Palestine | WVS 6 | 0.671 |  |  |  |  |  |
| Peru | WVS 6 | 0.722 | 2.78 | 4.11 | -1.06 | 48.7 | 368 |
| Philippines | WVS 6 | 0.651 | 2.89 | 3.96 | -0.15 | 45.3 |  |
| Poland | WVS 6 | 0.826 | 3.28 | 4.51 | -0.12 | 56.3 | 501 |
| Portugal | EVS | 0.805 | 3.56 | 4.38 | 0.60 | 56.7 | 490 |
| Qatar | WVS 6 | 0.847 | 3.55 | 5.10 | 0.52 | 55.9 | 373 |
| Romania | WVS 6 | 0.779 | 3.22 | 4.08 | -0.29 | 53.0 | 426 |
| Russia | WVS 6 | 0.773 | 3.03 | 4.24 | -0.09 | 52.8 | 468 |
| Rwanda | WVS 6 | 0.453 |  | 4.00 |  | 43.2 |  |
| Serbia | EVS | 0.743 | 2.68 | 3.84 |  |  | 442 |
| Singapore | WVS 6 | 0.894 | 4.65 | 5.48 | 2.45 | 76.7 | 543 |
| Slovak Republic | EVS | 0.824 | 3.48 | 4.25 | 0.27 | 56.7 | 488 |
| Slovenia | WVS 6 | 0.873 | 3.8 | 4.42 | 0.37 | 59.1 | 499 |
| South Africa | WVS 5 | 0.608 | 3.24 | 4.32 | 0.33 | 53.2 |  |
| South Korea | WVS 6 | 0.882 | 4.24 | 4.93 | 2.26 | 72.1 | 541 |
| Spain | WVS 6 | 0.864 | 3.74 | 4.49 | 0.93 | 58.8 | 484 |
| Sweden | WVS 6 | 0.895 | 4.85 | 5.56 | 1.64 | 80.3 | 495 |
| Switzerland | EVS | 0.903 | 4.82 | 5.63 | 2.23 | 78.1 | 517 |
| Taiwan | WVS 6 | 0.690 | 3.97 | 5.21 |  | 72.5 | 519 |
| Thailand | WVS 5 | 0.685 | 3.06 | 4.51 | 0.12 | 54.8 | 422 |
| Trinidad and Tobago | WVS 6 | 0.764 | 3.15 | 3.97 | -0.12 | 47.7 | 413 |
| Tunisia | WVS 6 | 0.715 | 3.05 | 4.65 | 0.14 | 54.1 | 392 |
| Turkey | WVS 6 | 0.738 | 2.99 | 4.25 | -0.21 | 50.2 | 455 |
| Ukraine | WVS 6 | 0.726 | 3.06 | 3.9 | -0.45 | 50.4 |  |
| United States | WVS 6 | 0.908 | 4.57 | 5.43 | 1.80 | 74.8 | 496 |
| Uruguay | WVS 6 | 0.779 | 3.17 | 4.23 | -0.95 | 52.8 | 427 |
| Uzbekistan | WVS 6 | 0.648 |  |  |  |  |  |
| Vietnam | WVS 5 | 0.598 | 2.95 | 4.27 | -0.65 | 47.1 |  |
| Yemen | WVS 6 | 0.484 |  |  |  | 36.3 |  |
| Zambia | WVS 5 | 0.471 | 2.63 | 3.55 | -1.28 | 42.5 |  |
| Zimbabwe | WVS 6 | 0.459 | 2.24 | 3.03 | -1.63 | 29.6 |  |

Note. Wave = data from representative samples administered in World Values Survey (WVS) and European Values Study (EVS), WVS 5 has been administered in 2005-2009, WVS 6 in 2010-2014, and EVS in 2008; HDI= Human Development Index; GII = Global Innovation Index; GCI= Global Competitiveness Index; ICI= Innovation Capacity Index; III= International Innovation Index; PISA = mean test score on mathematics, reading abilities and scientific knowledge.

**Main Statistical Analyses**

Analyses have been conducted in MPlus^2^. The code used for the analysis reported in Figure 2 is as follows:

TITLE: 1-1-2 INNOV (MSEM)

DATA: FILE IS Education.dat;

VARIABLE: NAMES ARE

cntry edu libval hdi eduhdi innov pisa;

MISSING ARE ALL (-99);

USEVARIABLES ARE

cntry edu libval hdi eduhdi innov pisa;

BETWEEN ARE hdi innov pisa;

CLUSTER IS cntry;

ANALYSIS: TYPE IS TWOLEVEL RANDOM;

MODEL: %WITHIN%

libval ON edu (a);

libval ON eduhdi (c);

%BETWEEN%

libval ON hdi (b);

innov ON edu;

innov ON hdi;

innov ON eduhdi;

innov ON libval (d);

MODEL CONSTRAINT:

NEW(ad);

ad = a*d;

NEW(bd);

bd = b*d;

NEW(cd);

cd = c*d;

OUTPUT: TECH1 TECH8 CINTERVAL;

The code for the analysis which corrects for human capital is as follows:

TITLE: 1-1-2 INNOV (MSEM)

DATA: FILE IS Education.dat;

VARIABLE: NAMES ARE

cntry edu libval hdi eduhdi innov pisa;

MISSING ARE ALL (-99);

USEVARIABLES ARE

cntry edu libval hdi eduhdi innov pisa;

BETWEEN ARE hdi innov pisa;

CLUSTER IS cntry;

ANALYSIS: TYPE IS TWOLEVEL RANDOM;

MODEL: %WITHIN%

libval ON edu (a);

libval ON eduhdi (c);

%BETWEEN%

libval ON pisa;

libval ON hdi (b);

innov ON edu;

innov ON hdi;

innov ON eduhdi;

innov ON libval (d);

innov ON pisa;

MODEL CONSTRAINT:

NEW(ad);

ad = a*d;

NEW(bd);

bd = b*d;

NEW(cd);

cd = c*d;

OUTPUT: TECH1 TECH8 CINTERVAL;

**Analysis of secular-rational values**

Table 1. Unstandardized (standard errors in brackets) and standardized estimates of multilevel regression analyses, examining the moderating role of the Human Development Index (HDI) in the association between education and secular-rational values

|  | *Secular-rational*  values | |
| --- | --- | --- |
|  | *b* (SE) | *β* |
| Education | 0.09 (0.01) | 0.13*** |
| HDI | 1.74 (0.22) | 0.39*** |
| Education X HDI | 0.34 (0.05) | 0.08*** |

Note: *: p < .05; **: p < .01; ***: p < . 001

Figure 1. Cross-level interactions between education and the Human Development Index on secular-rational values


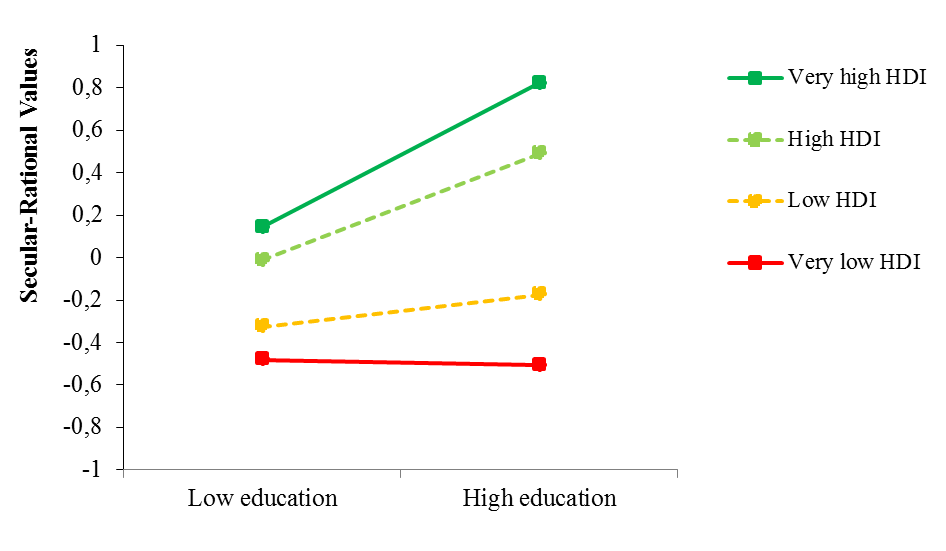


**Analysis of self-expression values**

Table 2. Unstandardized (standard errors in brackets) and standardized estimates of multilevel regression analyses, examining the moderating role of the Human Development Index (HDI) in the association between education and self-expression values

|  | *Self-expression*  values | |
| --- | --- | --- |
|  | *b* (SE) | *β* |
| Education | 0.14 (0.01) | 0.22*** |
| HDI | 1.33 (0.20) | 0.33*** |
| Education X HDI | 0.33 (0.05) | 0.08*** |

Note: *: p < .05; **: p < .01; ***: p < . 001

Figure 2. Cross-level interactions between education and the Human Development Index on secular-rational values (left panel), on self-expression values (middle panel), and on aggregated liberal values (right panel)


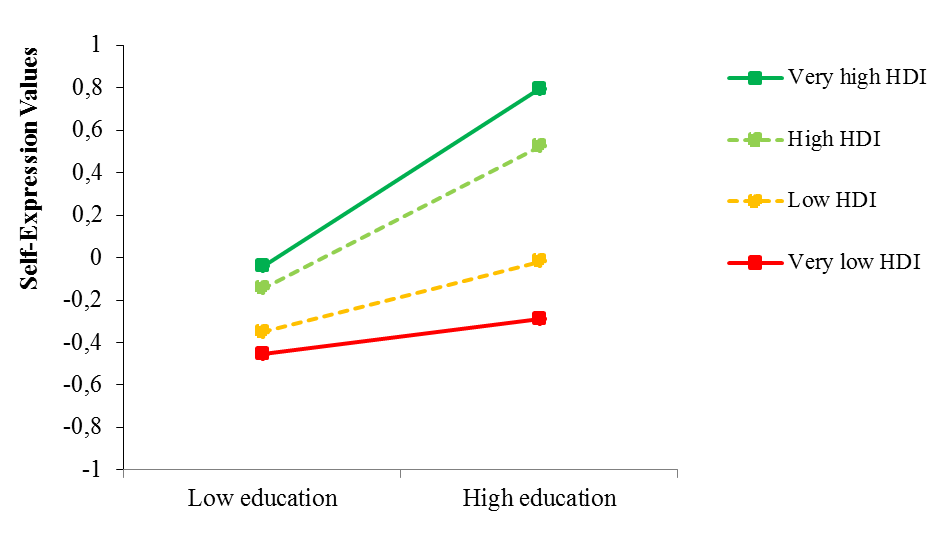


**References**

1. WVS Wave 6 (2014) World Values Survey Wave 6. (World Values Survey Association, Aggregate File Producer: Asep/JDS, Madrid, Spain).
2. ESS Round 4 (2008) European Social Survey Round 4 Data. (Norwegian Social Science Data Services, Norway – Data Archive and Distributor of ESS data).
3. Inglehart, R (1990) *Culture shift in advanced industrialized society*. (Princeton, NJ: Princeton University Press).
4. Inglehart, R, Welzel, C (2005) *Modernization, cultural change, and democracy. The human development sequence*. (New York: Cambridge University Press)
5. Welzel, C (2013) *Freedom rising. Human empowerment and the quest for emancipation.* (New York: Cambridge University Press)
6. Tavakol M, Dennick, R (2011) Making sense of Cronbach’s alpha. *Int J Med Educ*, 2: 23-55.
7. United Nations Development Program, Retrieved 30 April 2015. <http://hdr.undp.org/en/humandev>
8. Dutta, S (Ed) (2010), *The Global Innovation Index* 2009-2010 (Fontainebleau, France, INSEAD)
9. Schwab, K (Ed) (2010), *The Global Competitiveness Index Report 2010-2011* (Geneva, Switzerland, World Economic Forum)
10. Lopez-Claros, A (Ed), The innovation for development report 2010-2011 (London, UK, Palgrave Macmillan, 2011)
11. Andrew, JP, DeRocco, ES, Taylor, A (2009) The innovation imperative in manufacturing (Boston, The Boston Consulting Group Inc).
12. OECD (2010) *PISA 2009 Results,* http://dx.doi.org/10.1787/9789264091580-en.
13. Muthén, LK, Muthén, BO (2012) *Mplus User’s Guide. Seventh Edition*. (Los Angeles, CA, Muthén & Muthén).
